# Garvicin KS, a Broad-Spectrum Bacteriocin Protects Zebrafish Larvae against *Lactococcus garvieae* Infection

**DOI:** 10.3390/ijms23052833

**Published:** 2022-03-04

**Authors:** Saurabh Dubey, Dzung B. Diep, Øystein Evensen, Hetron M. Munang’andu

**Affiliations:** 1 Department of Paraclinical Sciences, Faculty of Veterinary Medicine, Norwegian University of Life Sciences, P.O. Box 5003, 1433 Ås, Norway; saurabh.dubey@nmbu.no (S.D.); oystein.evensen@nmbu.no (Ø.E.); 2 Department of Production Medicine, Faculty of Veterinary Medicine, Norwegian University of Life Sciences, P.O. Box 5003, 1433 Ås, Norway; 3 Faculty of Chemistry, Biotechnology and Food Science, Norwegian University of Life Sciences, 1433 Ås, Norway; dzung.diep@nmbu.no; 4 Faculty of Biosciences and Aquaculture, Nord University, 8049 Bodø, Norway

**Keywords:** GarKS, bacteriocin, inhibition, cytotoxicity, zebrafish, *Lactococcus garvieae*

## Abstract

Bacteriocins are emerging as a viable alternative to antibiotics due to their ability to inhibit growth or kill antibiotic resistant pathogens. Herein, we evaluated the ability of the bacteriocin Garvicin KS (GarKS) produced by *Lactococcus garvieae* KS1546 isolated from cow milk to inhibit the growth of fish and foodborne bacterial pathogens. We found that GarKS inhibited the growth of five fish *L. garvieae* strains isolated from infected trout and eels. Among fish pathogens, GarKS inhibited the growth of *Streptococcus agalactiae* serotypes Ia and Ib, and *Aeromonas hydrophila* but did not inhibit the growth of *Edwardsiella tarda*. In addition, it inhibited the growth of *A. salmonicida* strain 6421 but not *A. salmonicida* strain 6422 and *Yersinia ruckeri*. There was no inhibition of three foodborne bacterial species, namely *Salmonella enterica*, *Klebsiella pneumoniae*, and *Escherichia coli*. In vitro cytotoxicity tests using different GarKS concentrations showed that the highest concentration of 33 µg/mL exhibited low cytotoxicity, while concentrations ≤3.3 µg/mL had no cytotoxicity on CHSE-214 and RTG-2 cells. In vivo tests showed that zebrafish larvae treated with 33 µg/mL and 3.3 µg/mL GarKS prior to challenge had 53% and 48% survival, respectively, while concentrations ≤0.33 µg/mL were nonprotective. Altogether, these data show that GarKS has a broad inhibitory spectrum against Gram positive and negative bacteria and that it has potential applications as a therapeutic agent for a wide range of bacterial pathogens. Thus, future studies should include clinical trials to test the efficacy of GarKS against various bacterial pathogens in farmed fish.

## 1. Importance

Aquaculture is the fastest expanding food producing sector in the world. This rapid expansion has brought with it an increase in bacterial diseases, which has led to increased use of antimicrobials. This has led to increase in antimicrobial resistant (AMR) bacteria in farmed aquatic organisms, with substantial quantities of AMRs ending up in wild aquatic organisms and environments used for recreation. Thus, antimicrobial use in aquaculture is contributing to environmental expansion of AMR. To overcome this problem, there is a need to find alternative remedial compounds that do not pose the threat of drug resistance, having broad therapeutic spectra against different bacterial pathogens in aquaculture. It is for this reason that we examined the therapeutic properties of GarKS as an alternative to antibiotic use against fish pathogens. Our findings have significant implications on the use of bacteriocins as an alternative to antibiotics in aquaculture.

## 2. Introduction

Aquaculture is one of the fastest growing food producing sectors in the world [1] and bacterial diseases cause high economic losses in aquaculture. Although vaccine development has contributed to reducing the occurrence of bacterial diseases in aquaculture, there are still many pathogens that have not been successfully controlled through vaccination [2]. The use of antibiotics for the treatment of bacterial diseases poses the danger of selecting for antibiotic resistant bacteria. Thus, there is need for novel antimicrobials with broad therapeutic spectrums. Bacteriocins which are widely used in food preservation [3], have great potential as a source for antimicrobials to help reduce bacterial infections in aquaculture. Bacteriocins are ribosomally synthesized antibacterial peptides that either kill or inhibit the growth of closely related bacteria, to which the producer has a specific mechanism that prevents self-destruction [4,5]. Unlike antibiotics, most bacteriocins have a narrow antimicrobial inhibition spectrum, although the inhibition of food-borne zoonoses, food spoilage, and antimicrobial resistant (AMR) bacteria has been reported [6].

By 2014, more than 200 bacteriocins had been deposited in the open-access database (BACTIBASE) [7] and by 2019, more than 510 lactic acid bacteria (LAB) bacteriocins had been deposited in the LABiocin database [8]. Many bacteriocins have activity against foodborne and mammalian pathogens. Bacteriocins from *Vibrio mediterranei*, *Lactiplantibacillus plantarum*, and *Carnobcterium piscicola* have been shown to inhibit the growth of *Aeromonas hydrophila, Vibrio* spp. and *Pseudomonas* spp. that cause haemorrhagic septicaemia in farmed fish [9,10,11]. Given that most of these bacteriocins have a narrow antimicrobial spectrum, there is need to identify bacteriocins with broad activity. Recently, Ovchinnikov et al. [11] identified in raw milk samples from Kosovo a highly potent bacteriocin from *Lactococcus garvieae* KS1546, which is heat stable and proteinase-labile with an inhibition spectrum covering numerous distantly related genera, including several LAB and foodborne bacteria. The bacteriocin known as Garvicin KS (GarKS) belongs to a so-called multi-peptide leaderless bacteriocin group. It is composed of three peptides (GakA, GakB, and GakC) with a size between 30–34 aa, to make a functional unit, and these peptides are secreted by a dedicated ABC-transporter without involving a leader-peptide, hence the name leaderless bacteriocin [11]. It has also been shown to have inhibitory effects on pathogenic bacteria belonging to genera such as *Bacillus*, *Listeria*, *Streptococcus* and *Staphylococcus,* including methicillin resistant *Staphylococcus aureus* (MRSA) and vancomycin-resistant enterococci (VRE) [11,12,13]. However, its inhibitory properties have not been tested against farmed fish and foodborne pathogens. Moreover, there is need to test its cytotoxicity using in vitro cell-culture methods to determine its safety on host cells and to carry out in vivo test to determine its ability to protect fish exposed to different bacterial pathogens

*Lactococcus garvieae* is the etiological agent of lactococcosis, which is a hyperacute and hemorrhagic septicemia associated with high economic losses in aquaculture. It causes high mortalities, decreased growth rate and poor body conditions in several farmed aquatic species of commercial importance such as rainbow trout (*Oncorhynchus mykiss*), yellowtail (*Seriola quinqueradiata*), tilapia (*Oreochromis* sp.), Japanese eel (*Anguilla japonica*), olive flounder (*Paralichthys olivaceous*), grey mullet (*Mugil cephalus* L), amberjack (*Seriola dumerili*), and giant fresh water prawn (*Macrobrachium rosenbergii*) [14,15]. It is an emerging zoonotic pathogen due to increasing reports of human infections where it causes endocarditis, urinary tract infections, liver abscesses, septicemia, and peritonitis [16]. Infections in humans have been linked to consumption or handling of contaminated fish, meat, and milk [17].

The aim of the present study was to evaluate the inhibitory effects of GarKS on farmed fish and foodborne pathogens. We also wanted to determine its cytotoxicity on two cell lines derived from two different fish species and further assess its ability to protect zebrafish larvae (*Danio rerio*) against *L. garvieae* infection. We envisage that the data generated in this study will shed new insights on the protective ability of GarKS against fish pathogens.

## 3. Materials and Methods

### 3.1. Source of Antimicrobial Peptide Garvicin KS

Synthetic peptides GakA, GakB, and GakC, which constitute the bacteriocin GarKS, were synthesized by Pepmic Co., Ltd., Suzhou, Jiangsu, China, with >95% purity. The peptides were solubilized in 0.1% (*vol*/*vol*) trifluoracetic acid (TFA; Sigma-Aldrich).

### 3.2. GarKS Inhibition of Fish and Foodborne Bacterial Pathogens

The inhibitory properties of the synthesized GarKS bacteriocin was tested against different fish and foodborne bacterial pathogens. In addition, it was also tested against different fish *L. garvieae* strains. To do this, five fish pathogenic *L. garvieae* strains b. baraja, lg, and perbembe from infected farmed rainbow trout [18], cp-1 and BA063090 from infected trout and eel [19], as well as the GarKS producer *L. garvieae* KS1546 isolated from cow milk [20] (Table 1) were cultured in Mueller–Hinton broth at 30 °C overnight. In addition, fish pathogens *Streptococcus agalactiae* serotypes Ia and Ib from Nile tilapia (*Oreochromis niloticus*), *Aeromonas hydrophila* from rohu (*Labeo rohita*) and *Edwardsiella tarda* from Olive flounder (*Paralichthys olivaceus*) (Table 1) were grown at 37 °C while *Aeromonas salmonicida* 6421, *A. salmonicida* 6422 and *Yersinia ruckeri* were grown at 25 °C. After overnight culture, bacteria suspensions in Mueller–Hinton broth were centrifuged at 3000 rpm followed by discarding the supernatants. The pellets were washed twice using phosphate-buffered saline (PBS). Thereafter, 100 µL of 10^7^ CFU/mL bacterial suspension was spread on a Mueller–Hinton agar plate. Next, 50 µL of 33 µg/mL GarKS was put in the well at the centre of each plate followed by incubation at 30 °C for plates cultured with different *L. garvieae* strains, while plates cultured with fish pathogens were incubated at 37 °C and 25 °C as described above. Inhibition of bacterial growth by GarKS was measured after 24 h.

To determine the concentration of GarKS able to inhibit the growth of fish pathogens, *S. agalactiae* (serotype Ia and Ib), *A*. *salmonicida* 6421, *Y. ruckeri*, and *A. hydrophila* 19 were exposed to five different GarKS concentrations of 33 µg/mL, 3.3 µg/mL, 0.33 µg/mL, 0.033 µg/mL, 0.0033 µg/mL. Like the method above, 100 µL of 10^7^ CFU/mL bacterial suspension was spread on Mueller–Hinton agar plate, while 50 µL of different GarKS concentrations were put in five separate wells on each plate. Plates cultured with *A. hydrophila* 19 and *S. agalactiae* serotypes Ia and Ib were incubated at 37 °C, while plates cultured with *A. salmonicida* 6421 and *Y. ruckeri* were incubated at 25 °C. The inhibitory effect of the different concentrations of GarKS was measured after overnight culture.

To determine the inhibitory effects of GarKS on fish and foodborne bacterial pathogens grown at different temperatures, *Y. ruckeri*, *K. pneumoniae*, *E. coli*, and *S. enterica* were cultured by spreading 100 µL of 10^7^ CFU/mL bacterial suspension on Mueller–Hinton agar plate while 50 µL of 33 µg/mL GarKS were put in the wells at the centre of each plate, followed by incubation at 25 °C, 30 °C and 37 °C. The inhibitory effect of GarKS was measured after overnight culture.

### 3.3. Cytotoxicity Test of GarKS in Fish Cell Lines Based on LDH Test

Chinook salmon embryo 214 (CHSE-214) cells (ECACC 91041114) derived from Chinook salmon (*Oncorhynchus tshawytscha*) embryos and rainbow trout gill II (RTG-2) (ATCC CCL 55) cells derived from rainbow trout (*O. mykiss*) gonad tissues were used to determine the cytotoxicity of GarKS using the Pierce LDH Cytotoxicity Assay Kit (CyQUANT™ LDH, ThermoFisher Scientific, Waltham, MA, USA). Both CHSE-214 and RTG-2 cells were grown in 96 well plates at 20 °C in Leibovitz’s L-15 Medium (L-15) (Sigma-Aldrich) supplemented with 10% fetal bovine serum (FBS). When cells were about 80% confluent, the L-15 growth media was removed, followed by washing twice using PBS. Then, 100 µL GarKS was added to each well. Five concentrations (33 µg/mL, 3.3 µg/mL, 0.33 µg/mL, 0.033 µg/mL and 0.0033 µg/mL) were used on both cell lines. To evaluate the cytotoxicity of different GarKS concentrations on both cell lines cultured at different temperatures, GarKS treated CHSE-214 and RTG-2 cells in 96-well plates were incubated at 25 °C and 37 °C. After overnight incubation, 50 µL GarKS treated samples were transferred to new 96 well plates followed by adding 50 µL Pierce LDH cytotoxicity solution. The plates were incubated at room temperature for 30 min in a dark chamber. For the positive control, 50 µL lysis buffer were used while 50 µL PBS were used as a negative control. After adding 50 µL stop solution to each well, results were read by spectrophotometry (TECAN, Morrisville, NC, USA) at an optical density (OD) of 492 nm.

### 3.4. Infection Study of Zebrafish Larvae against Lactococcus garvieae

Zebrafish experiments were carried out based on ethics for short-term toxicity test procedures of the Fish Embryo Toxicity (FET) OECD Guidelines [21]. The species used was the zebrafish (*Danio rerio*) wild strain AB cultured at the wet laboratory at the Norwegian University of Life Sciences (NMBU). To determine the lethal dose 50 (LD_50_) of *L. garvieae* strain b. baraja in zebrafish larvae, the *L. garvieae* strain grown overnight at 30 °C in tryptose soy broth (TSB) medium was centrifuged at 3000 rpm for 15 min and washed twice using PBS. The bacterial concentration was determined using the CFU/mL method. Serially diluted bacteria from 10^−1^ to 10^−10^ were used to determine the lethal dose 50 (LD_50_) concentration able to cause 50% mortality in zebrafish larvae. Ten zebrafish larvae were exposed to each bacterial concentration in Petri-dishes. Mortality was recorded after 24 h and used to calculate the LD_50_.

The LD_50_ concentration determined above was used to test the ability of GarKS to prevent mortality in zebrafish larvae challenged with *L. garvieae* b. baraja. A total of 210 healthy zebrafish larvae selected 48 h post fertilization (hpf) were randomly assigned into six groups each having 30 larvae. Five groups were exposed to different GarKS concentrations for 3 h of 33 µg/mL, 3.3 µg/mL, 0.33 µg/mL, 0.033 µg/mL and 0.0033 µg/mL prior to challenge. Each group was assigned 30 zebrafish larvae while the noninfected negative control group was exposed to PBS. Challenge was done in triplicates of which the 30 zebrafish larvae assigned to each group were put in batches of 10 larvae per Petri-dish. After exposure to GarKS was completed, zebrafish larvae were transferred to new Petri dishes and bath challenged with 10^8^ CFU/mL *L. garvieae* (determined by the LD_50_ method above). The larvae were exposed (continuously) to the pathogen solution until final sampling (96 h post challenge) and mortality was recorded every 6 h post challenge (hpc) with final recording at 96 hpc.

### 3.5. Statistical Analyses

All data were transferred to GraphPad Prism version 9 (www.graphpad.com, accessed on 3 March 2022) for statistical analysis. The Kaplan Meyer’s survival analysis was used to determine the post challenge survival proportions (PCSP) in larvae challenged with *L. garvieae* using different GarKS concentration. The student’s *t*-test was used to compare differences between groups. Difference between groups were considered significant for *p* < 0.05 (95% confidence interval).

## 4. Results

### 4.1. Inhibition of Fish Pathogens by GarKS

**Figure 1:**
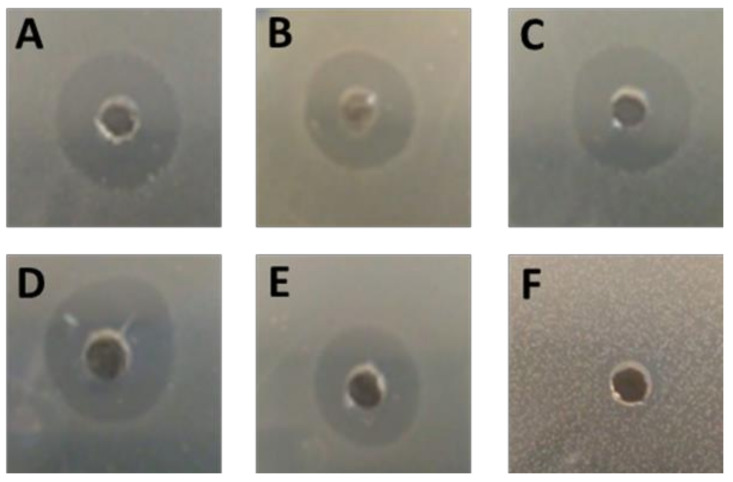
Inhibition of *Lactococcus garvieae* growth by GarKS cultured overnight on Mueller–Hinton agar plates at 30 °C. Note the inhibition zones around *L. garvieae* strain (**A**) b. baraja, (**B**) lg, (**C**) perbembe, (**D**) cp-1, and (**E**) Ba063090, all from infected fish. Additionally, note the absence of inhibition zone around strain KS2546 (**F**) which is the bacteriocin producer itself. For all *L. garvieae* strains, 100 µL of 10^7^ CFU/mL bacterial suspension were spread on Mueller–Hinton agar plate, while 50 µL of 33 µg/mL GarKS were put in the well at the centre of each plate.

**Figure 2:**
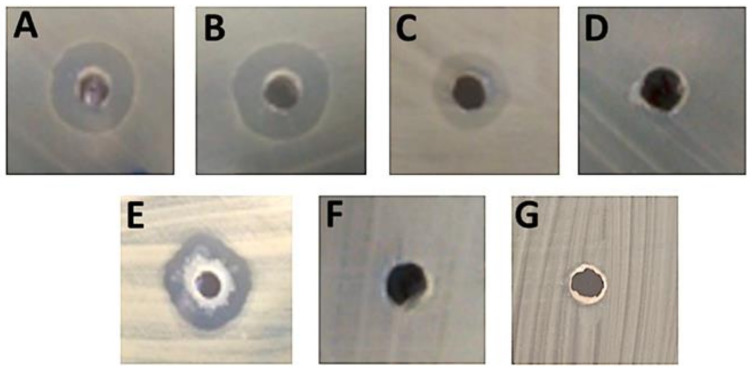
Inhibition of bacterial pathogens of fish species by GarKS cultured overnight on Mueller–Hinton agar plates. (**A**) Inhibition zone around *Streptococcus agalactiae* serotype Ia isolated from Nile tilapia (*Oreochromis niloticus*) in Taiwan, (**B**) inhibition zone around *S. agalactiae* serotype Ib from Nile tilapia in Taiwan, (**C**) inhibition zone around *Aeromonas hydrophila* strain 19 from rohu (*Labeo rohita*) in India and (**D**) no inhibition zone around *Edwardsiella tarda* from Olive flounder (*Paralichthys olivaceus*) in South Korea. (**E**) Note the presence of inhibition zone around *Aeromonas salmonicida* strain 6421 and the absence of inhibition zones around (**F**) *A. salmonicida* strain 6422 and (**G**) *Yersinia ruckeri* isolated from Atlantic salmon (*Salmo salar* L). *S. agalactiae*, *A. hydrophila* and *E. tarda* were cultured at 37 °C while *A. salmonicida* and *Y. ruckeri* were cultured at 25 °C. For all bacterial species, 100 µL of 10^7^ CFU/mL bacterial suspension were spread on Mueller–Hinton agar plate while 50 µL of 33 µg/mL GarKS were put in the well at the centre of each plate.

**Figure 3:**
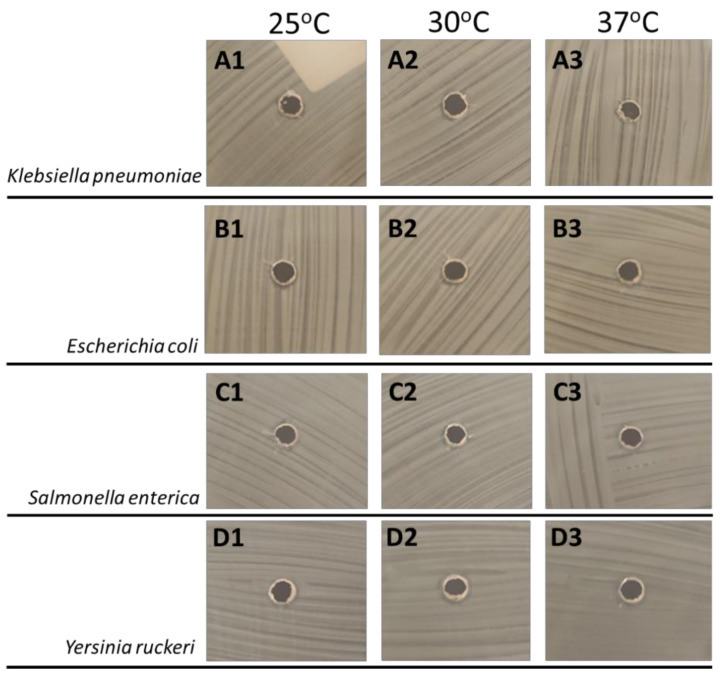
Lack of inhibition of fish and foodborne bacterial pathogens cultured overnight on Mueller–Hinton agar plates at 25 °C, 30 °C and 37 °C., *Klebsiella pneumoniae* (**A1**–**A3**), *Escherichia coli* (**B1**–**B3**), and *Salmonella enterica* (**C1**–**C3**) *Yersinia ruckeri* (**D1**–**D3**) showing no inhibition at 33 µg/mL GarKS after overnight culture on Mueller–Hinton agar plates at 25 °C, 30 °C and 37 °C.

Our findings show that the growth of all five fish *L. garvieae* strains b. baraja, lg, perbembe, cp-1, and BA063090 was inhibited by GarKS, as shown by clear inhibition zones on Mueller–Hinton agar plates (Figure 1A–E). All fish-derived *L. garvieae* strains had equal diameters of inhibition zones around GarKS at 33 µg/mL on the growth agar plates after overnight incubation at 30 °C (Table 1). On the contrary, the *L. garvieae* strain KS1546 which is the GarKS producer itself was not inhibited by GarKS shown by lack of inhibition zone on the growth agar plate (Figure 1F). For fish pathogens, *S. agalactiae* serotypes Ia and Ib were inhibited by 33 µg/mL GarKS after overnight incubation at 37 °C (Figure 2A,B), while growth of *A. hydrophila* 19 was partially inhibited (Figure 2C) and *E. tarda* growth was not inhibited at all by GarKS after overnight culture at 37 °C (Figure 2D). The growth of *A. salmonicida* strain 6421 was inhibited (Figure 2E), while *A. salmonicida* strain 6422 (Figure 2F) and *Y. ruckeri* (Figure 2G) were not inhibited by 33 µg/mL GarKS after overnight incubation on Mueller–Hinton agar at 25 °C. Equally, *Y. ruckeri* was not inhibited by GarKS after overnight culture at three temperatures of 25 °C, 30 °C and 37 °C (Figure 3D).

### 4.2. Inhibition of Foodborne Pathogens by GarKS

Our findings show that all foodborne bacterial pathogens, *K. pneumoniae*, *E. coli*, and *S. enterica* were not inhibited at 33 µg/mL GarKS at all three temperatures of 25 °C, 30 °C and 37 °C after overnight culture (Figure 3A–C).

### 4.3. Inhibition of Bacterial Fish Pathogens at Different GarKS Concentrations

**Figure 4:**
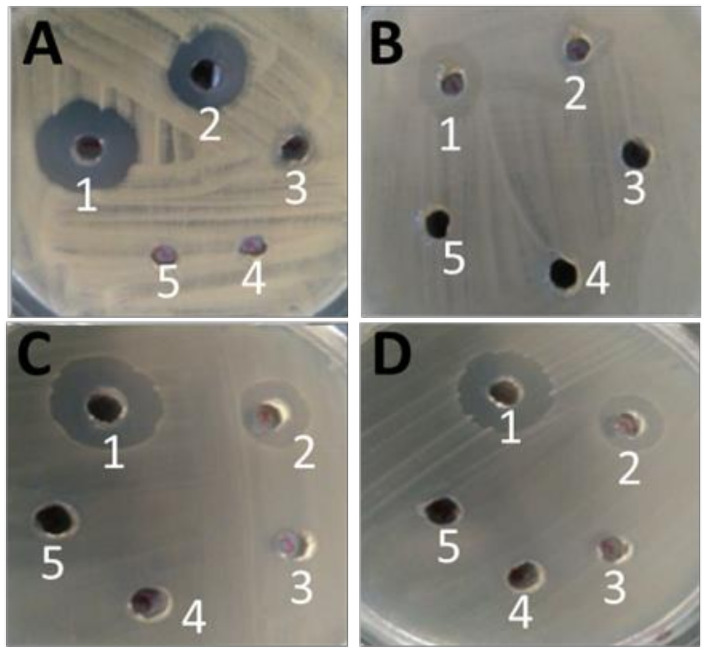
Inhibition of bacterial pathogens isolated from fish exposed to five different GarKS concentrations of 33 µg/mL (1), 3.3 µg/mL (2), 0.33 µg/mL (3), 0.033 µg/mL (4) and 0.0033 µg/mL (5). (**A**) Note inhibition of *Aeromonas salmonicida* strain 6421 at high concentrations of 33 µg/mL and 3.3 µg/mL, and no inhibition around low concentrations ≤0.33 µg/mL. (**B**) Inhibition around *A. hydrophila* for higher concentrations of 33 µg/mL and 3.3 µg/mL, and no inhibition around low concentrations ≤0.33 µg/mL GarKS. (**C**) Inhibition of *Streptococcus agalactiae* serotype Ia for high concentration of 33 µg/mL and 3.3 µg/mL and no inhibition on low concentrations ≤0.33 µg/mL GarKS. (**D**) Inhibition of *S. agalactiae* serotype Ib high concentrations of 33 µg/mL and 3.3 µg/mL and no inhibition on low concentrations ≤0.33 µg/mL GarKS.

GarKS inhibited the growth of *A. salmonicida* 6421 at concentrations of 33 µg/mL and 3.3 µg/mL, while a partial inhibition was seen for the 0.33 µg/mL concentration and no inhibition for lower concentrations (0.33 µg/mL–0.0033 µg/mL) was seen on Mueller–Hinton agar after overnight culture at 25 °C (Table 2, Figure 4A). Equally, the growth of *S. agalactiae* serotype Ia and Ib was inhibited at high concentrations, 33 µg/mL and 3.3 µg/mL, but no inhibition was observed for the lower concentrations ≤0.33 µg/mL after overnight culture at 37 °C (Table 2, Figure 4C,D). There was low inhibition of *A. hydrophila* strain 19 at 33 µg/mL and 3.3 µg/mL, while concentrations <3.3 µg/mL showed no growth inhibition of *A. hydrophila* 19 after overnight culture at 37 °C (Figure 4B).

### 4.4. Cytotoxicity of GarKS on CHSE-214 and RTG-2 Cells

**Figure 5:**
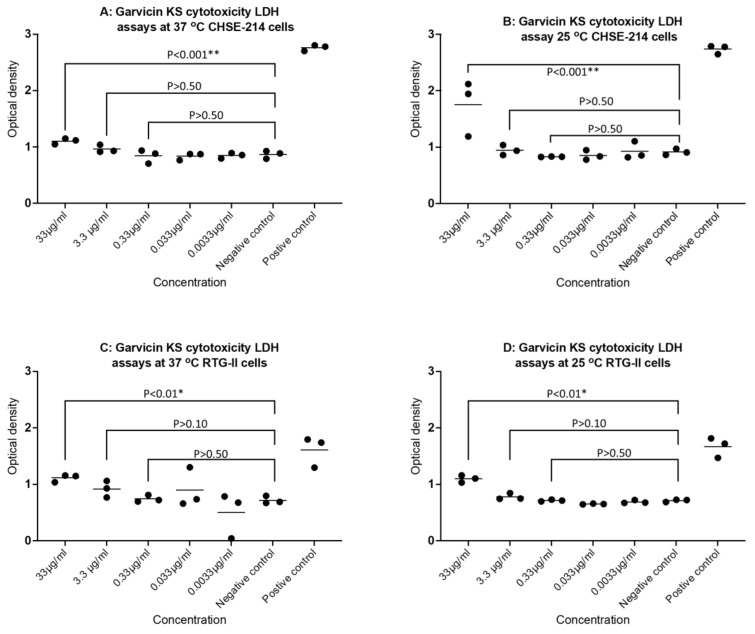
Cytotoxicity LDH test of GarKS on Chinook salmon embryo 214 (CHSE-214) and rainbow trout gill II (RTG-2) cell exposed to concentrations of 33 µg/mL, 3.3 µg/mL, 0.33 µg/mL, 0.033 µg/mL, and 0.0033 µg/mL GarKS cultured at 37 °C and 25 °C. Negative control represents cells exposed to PBS while positive control represents cells treated with the LDH solution based on manufacturers’ guidelines (CyQUANT™ LDH, ThermoFisher Scientific, Waltham, MA, USA). Upper channel shows GarKS treated CHSE 214 cells cultured at 37 °C (**A**) and 25 °C (**B**) while the lower channel shows GarKS treated RTG-2 cells cultured at 37 °C (**C**) and 25 °C (**D**).

Cytotoxicity based on the LDH assay was only observed at the highest concentration, 33 µg/mL GarKS in CHSE-214 cells cultured at 25 °C and 37 °C. That said, leakage of LDH in CHSE-214 cells exposed to 33 µg/mL GarKS was significantly lower (*p* < 0.001) than in cells treated with the positive control LDH solution (Figure 5A,B). There was indication of induced cytotoxicity in CHSE-214 cells treated with lower concentrations of GarKS (≤3.3 µg/mL), cultured at 25 °C and 37 °C (Figure 5A,B). For RTG-2 cells, cytotoxicity was only observed in cells treated with the highest concentration of 33 µg/mL GarKS cultured at 25 °C and 37 °C (*p* < 0.01) (Figure 5C,D), and again levels of leakage were significantly lower (*p* < 0.0001) than in cells treated with the LDH positive control solution (Figure 5C,D). No cytotoxicity was observed in RTG-2 cells treated with concentrations ≤3.3 µg/mL GarKS cultured at 25 °C and 37 °C (Figure 5C,D).

### 4.5. Kaplan Meyer’s Survival Analysis of Zebrafish Larvae Challenged with L. garvieae

**Figure 6:**
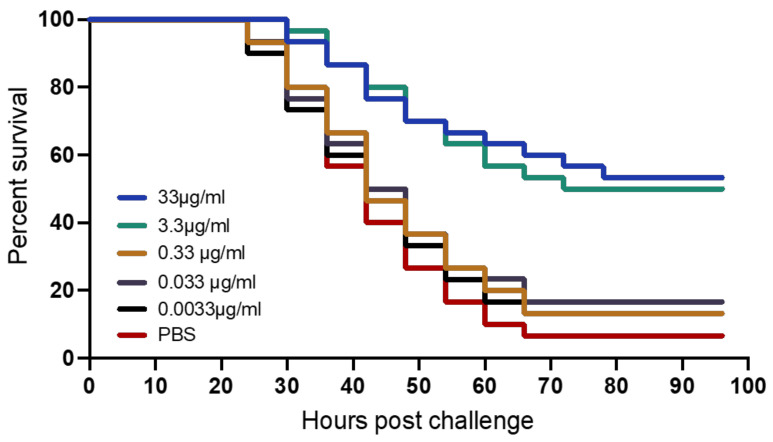
Kaplan Meyer’s post challenge survival analysis of zebrafish larvae challenged with *Lactococcus garvieae* after treatment with five different concentrations (33 µg/mL, 3.3 µg/mL, 0.33 µg/mL, 0.033 µg/mL, and 0.0033 µg/mL) of GarKS.

The Kaplan Meyer’s survival analysis showed the survival plots over a 96-h observation period for zebrafish larvae, following lethal challenge with *L. garvieae* (10^8^ CFU/mL). Zebrafish larvae pre-treated with the highest concentration, 33 µg/mL GarKS, had a survival of 53% and with 48% survival for the 3.3 µg/mL GarKS-treated group (Figure 6). There was no difference in mortality for the two doses (*p* = 0.8221). Mortalities in both groups were significantly higher (*p* = 0.0001) than the mortality in noninfected larvae (treated with PBS). Larvae pre-treated with concentrations of 0.33 µg/mL, 0.033 µg/mL and 0.0033 µg/mL had survival of 13.33%, 16.66% and 13.33%, respectively, which was not significantly different (*p* ≥ 0.25) for any of the groups compared with the noninfected negative control group. This shows that zebrafish pre-treated with GarKS at 33 µg/mL and 3.3 µg/mL gave significantly increased survival following lethal challenge with *L. garvieae.*

## 5. Discussion

In this study, we have shown the inhibitory properties of GarKS on growth of different fish bacterial pathogens, foodborne bacteria, and its protective ability against lethal challenge with *L. garvieae* in zebrafish larvae.

Chi and Holo [22] showed that GarKS inhibited the growth of *Acinetobacter* but not the growth of other Gram-negative bacteria. Equally, we found that GarKS did not inhibit the growth of Gram-negative foodborne bacteria *S. enterica*, *K. pneumoniae*, and *E. coli*. Yet, GarKS inhibited the growth of Gram-negative, fish pathogenic *A. salmonicida* strain 6421 and *A. hydrophila*. GarKS also inhibited the growth of two Gram-positive *S. agalactiae* serotypes characterized as Ia and Ib, based on differences in the capsular antigens [23]. Altogether, our findings show that GarKS has inhibitory properties against bacterial pathogens of different fish species, pointing to its potential application as a broad-spectrum therapeutic agent against a wide range of bacterial pathogens in aquaculture. In addition, the inhibitory properties of GarKS seen against fish pathogens such as *S. agalactiae* and *A. hydrophila,* where both are potential human pathogens [24,25,26], suggest that GarKS has potential application as a therapeutic agent against fish-borne zoonoses in humans.

Inhibitory effects of Garvicin bacteriocins against other *L. garvieae* strains have been reported previously by different scientists [27,28,29,30]. For example, the bacteriocin Garvicin Q (GarQ) produced by *L. garvieae* BCC 43578 isolated from fermented pork sausage [29] was shown to inhibit the growth of other *L. garvieae* strains isolated from cow milk [30] and mallard ducks (*Anas platyrhynchos*) [27]. Similarly, Garvicin A (GarA) isolated from a human clinical case of *L. garvieae* 21881 infection, was shown to inhibit other *L. garvieae* strain including fish and bovine-pathogenic isolates [28]. *L. garvieae* DCC43, isolated from mallard ducks had inhibitory properties against fish *L. garvieae* strains CECT 5807, 5806, and 5274 [27]. Likewise, our findings show that GarKS produced by the *L. garvieae* strain KS1546 from cow milk inhibited the growth of five fish *L. garvieae* strains, supporting previous observations that Garvicin bacteriocins have inhibitory properties against other *L. garvieae* strains from different host species. Given that diseases produced by pathogenic *L. garvieae* strains cause high economic losses in farmed fish, the use of GarKS as therapeutic agent would be highly beneficial in reducing economic losses caused by *L. garvieae* in aquaculture. Additionally, GarKS would be beneficial as a therapeutic agent against the increasing *L. garvieae* infections in humans.

Our findings showed low cytotoxicity in both CHSE-214 and RTG-2 cells treated at highest concentration of GarKS and at lower concentrations (≤3.3 µg/mL), cytotoxicity was not observed. In vitro cytotoxicity tests are widely used as the first step to evaluate the biological safety of different chemical compounds used for drug development. Likewise, several bacteriocins have been tested using cytotoxicity assays prior to use as therapeutic agents. Dicks et al. [31] pointed out that most bacteriocins have low or no cytotoxicity on different host cells, while Maher and McClean [32] found that bacteriocins are cyto-neutral towards different eukaryotic cells, even at doses 100-fold higher than saturated killing concentrations [33]. For example, garvicin Q was shown not to be cytotoxic to Vero cells [29], while a bacteriocin purified from *L. garvieae* subsp. *bovis* BSN307 did not cause cytotoxicity in H9c2 cells [34]. Contrary to this, some studies have reported cytotoxicity of bacteriocins such as cytolysins and microcin E492 in mammalian cells [35,36]. Thus, the cytotoxicity of each single bacteriocin should be determined before considering its use as a remedial compound. These findings suggest that GarKS might have low or no adverse effects in fish when used as a therapeutic agent.

In this study, 33 µg/mL and 3.3 µg/mL GarKS concentrations produced 53.33% and 47.67% survival after challenge with *L. garvieae*, respectively, while concentrations ≤0.33 µg/mL were nonprotective in zebrafish larvae. The use of in vivo animal models for testing novel drug efficacy is a prerequisite for discovery of active ingredients/substances. Evaluating drug efficacy using mammalian models is considered costly and slow, while the use of zebrafish larvae produces rapid results with protective mechanisms comparable with other vertebrates [37,38,39]. Kalyanasundaram et al. [38] reported 50% protection in zebrafish larvae treated with the LAB strain BLN34 bacteriocins from cow milk after challenge using *Mycobacterium kansasii,* while Ravindran et al. [37] reported 100% protection in zebrafish larvae treated with *Bacillus subtilis* bacteriocins [40] followed by challenge with *Vibrio cholerae*. Zebrafish larvae treated with the peocin bacteriocin had survival rates of 63.3% and 71.67% after challenge with *A. hydrophila* [39]. These findings suggest that GarKS has the potential to serve as a therapeutic agent against *L. garvieae* infections in farmed aquatic organisms in aquaculture. Moreover, zebrafish has increasingly become a valuable model of translational research used for testing the safety of novel drugs for humans [41,42,43] because of a relatively high homology with the human genome [44]. Therefore, these findings have significant public health implications, suggesting that GarKS has the potential to be used as a therapeutic agent against *L. garvieae* infections in humans, which has emerged to be an important zoonoses causing endocarditis and other clinical conditions in humans [16].

In summary, this study has shown that GarKS has broad antimicrobial spectrum against Gram negative and positive bacterial fish pathogens, despite not having inhibitory properties against the examined foodborne bacterial pathogens. Our findings show low GarKS in vitro cytotoxicity in fish cells at high concentration and we also showed protection in zebrafish larvae after challenge with *L. garvieae*. Altogether, our findings suggest that GarKS has potential applications as a therapeutic agent against a wide range of bacterial pathogens. We suggest that future studies involve clinical trials to test the efficacy of GarKS as a therapeutic agent for different bacterial fish diseases.

## Figures and Tables

**Table 1:** Bacterial isolates used for bacteriocins inhibition study.

List of Bacteria	Strain/ Serotype	Source of Isolate	Inhibition Zone Diameter (mm)				Growth Temp (°C)	Susceptibility to GarKS
			Plate-1	Plate-2	Plate-3	Mean		
*Lactococcus garvieae*	b. baraja	Trout	19	18.5	19	18.83	30	Yes
*Lactococcus garvieae*	lg	Trout	18	18	18	18.00	30	Yes
*Lactococcus garvieae*	perbembe	Trout	18	19	18.5	18.50	30	Yes
*Lactococcus garvieae*	cp-1	Trout	19	18.5	19	18.83	30	Yes
*Lactococcus garvieae*	BA063090	Eel	18	18.5	18	18.17	30	Yes
*Lactococcus garvieae*	KS1546	From cow milk	0	0	0	0.00	30	No
*Streptococcus agalactiae*	bio I	Tilapia	16.5	16	17	16.50	37	Yes
*Streptococcus agalactiae*	bio II	Tilapia	17.8	17	17.5	17.43	37	Yes
*Aeromonas salmonicida*	6421	Atlantic salmon	19	19.5	19	19.17	25	Yes
*Aeromonas salmonicida*	6422	Atlantic salmon	8.5	8.3	8.5	8.43	25	Yes (partial)
*Aeromonas hydrophila*	19	Labeo rohita	9.5	9.0	9.3	9.40	37	Yes (slight)
*Edwardsiella tarda*	FC05	Olive flounder	0	0	0	0	37	No
*Yersinia ruckeri*	NVS	Atlantic salmon	0	0	0	0	25	No
*Klebsiella pneumoniae*	13883	ATCC	0	0	0	0	37	No
*Escherichia coli*	25922	ATCC	0	0	0	0	37	No
*Salmonella enterica*	4931	ATCC	0	0	0	0	37	No

**Table 2:** Inhibition of different bacterial species exposed to different concentrations of GarKS bacteriocin.

Microorganism	Incubation Temperature on Mueller–Hinton Agar	GarKS Concentration and Inhibition Zone Diameter in mm				
		33 µg/mL	3.3 µg/mL	0.33 µg/mL	0.033 µg/mL	0.0033 µg/mL
*Aeromonas salmonicida* strain 6421	25 °C	19.0	10.0	0	0	0
*Aeromonas hydrophila* strain 19	37 °C	14.5	6.7	0	0	0
*Streptococcus agalactiae* serotype Ia	37 °C	18.5	9	0	0	0
*Streptococcus agalactiae* serotype Ib	37 °C	18.0	8.7	0	0	0

## Data Availability

Not applicable.

## References

[1] FAO (2012). The State of World Fisheries and Aquaculture, Opportunities and Challenges.

[2] Munang’andu H.M. (2018). Intracellular Bacterial Infections: A Challenge for Developing Cellular Mediated Immunity Vaccines for Farmed Fish. Microorganisms.

[3] O’Connor P.M., Kuniyoshi T.M., Oliveira R.P., Hill C., Ross R.P., Cotter P.D. (2020). Antimicrobials for food and feed; a bacteriocin perspective. Curr. Opin. Biotechnol..

[4] Karpinski T., Szkaradkiewicz A., Caballero B., Finglas P., Toldrá F. (2016). Encyclopedia of Food and Health.

[5] Cotter P., Hill C., Ross R. (2005). Bacteriocins: Developing innate immunity for food. Nat. Rev. Genet..

[6] Heilbronner S., Krismer B., Brötz-Oesterhelt H., Peschel A. (2021). The microbiome-shaping roles of bacteriocins. Nat. Rev. Genet..

[7] Hammami R., Zouhir A., Le Lay C., Ben Hamida J., Fliss I. (2010). BACTIBASE second release: A database and tool platform for bacteriocin characterization. BMC Microbiol..

[8] AL Kassaa I., Rafei R., Moukhtar M., Zaylaa M., Gharsallaoui A., Asehraou A., El Omari K., Shahin A., Hamze M., Chihib N.-E. (2019). LABiocin database: A new database designed specifically for Lactic Acid Bacteria bacteriocins. Int. J. Antimicrob. Agents.

[9] Carraturo A., Raieta K., Ottaviani D., Russo G.L. (2006). Inhibition of Vibrio parahaemolyticus by a bacteriocin-like inhibitory substance (BLIS) produced by *Vibrio mediterranei* 1. J. Appl. Microbiol..

[10] Bhugaloo-Vial P., Dousset X., Metivier A., Sorokine O., Anglade P., Boyaval P., Marion D. (1996). Purification and amino acid sequences of piscicocins V1a and V1b, two class IIa bacteriocins secreted by *Carnobacterium piscicola* V1 that display significantly different levels of specific inhibitory activity. Appl. Environ. Microbiol..

[11] Ovchinnikov K.V., Chi H., Mehmeti I., Holo H., Nes I.F., Diep D.B. (2016). Novel Group of Leaderless Multipeptide Bacteriocins from Gram-Positive Bacteria. Appl. Environ. Microbiol..

[12] Kranjec C., Kristensen S.S., Bartkiewicz K.T., Brønner M., Cavanagh J.P., Srikantam A., Mathiesen G., Diep D.B. (2021). A bacteriocin-based treatment option for *Staphylococcus haemolyticus* biofilms. Sci. Rep..

[13] Kranjec C., Ovchinnikov K.V., Grønseth T., Ebineshan K., Srikantam A., Diep D.B. (2020). A bacteriocin-based antimicrobial formulation to effectively disrupt the cell viability of methicillin-resistant *Staphylococcus aureus* (MRSA) biofilms. Npj Biofilms Microbiomes.

[14] Meyburgh C.M., Bragg R.R., Boucher C.E. (2017). Lactococcus garvieae: An emerging bacterial pathogen of fish. Dis. Aquat. Org..

[15] Vendrell D., Balcázar J.L., Ruiz-Zarzuela I., de Blas I., Gironés O., Múzquiz J.L. (2006). *Lactococcus garvieae* in fish: A review. Comp. Immunol. Microbiol. Infect. Dis..

[16] Choksi T.T., Dadani F. (2017). Reviewing the Emergence ofLactococcus garvieae: A Case of Catheter Associated Urinary Tract Infection Caused by Lactococcus garvieaeand Escherichia coli Coinfection. Case Rep. Infect. Dis..

[17] Gibello A., Galán-Sánchez F., Blanco M.D.M., Rodríguez-Iglesias M., Domínguez L., Fernandez-Garayzabal J.F. (2016). The zoonotic potential of *Lactococcus garvieae*: An overview on microbiology, epidemiology, virulence factors and relationship with its presence in foods. Res. Veter-Sci..

[18] Shahi N., Mallik S., Sahoo M., Chandra S., Singh A. (2018). First report on characterization and pathogenicity study of emerging Lactococcus garvieae infection in farmed rainbow trout, *Oncorhynchus mykiss* (Walbaum), from India. Transbound. Emerg. Dis..

[19] Aguado-Urda M., Rodríguez-Bertos A., Heras A.I.D.L., Blanco M.M., Acosta F., Cid R., Fernández-Garayzábal J.F., Gibello A. (2014). Experimental *Lactococcus garvieae* infection in zebrafish and first evidence of its ability to invade non-phagocytic cells. Veter-Microbiol..

[20] Hai C.I.M., Ovchinnikov K., Holo H., Ingolf F.N., Dzung B. (2017). Antimicrobial Peptide Produced by *Lactococcus garvieae* with a Broad Inhibition Spectrum World Academy of Science. Eng. Technol. Int. J. Agric. Biosyst. Eng..

[21] Sobanska M., Scholz S., Nyman A.-M., Cesnaitis R., Alonso S.G., Klüver N., Kühne R., Tyle H., De Knecht J., Dang Z. (2017). Applicability of the fish embryo acute toxicity (FET) test (OECD 236) in the regulatory context of Registration, Evaluation, Authorisation, and Restriction of Chemicals (REACH). Environ. Toxicol. Chem..

[22] Chi H., Holo H. (2018). Synergistic Antimicrobial Activity Between the Broad Spectrum Bacteriocin Garvicin KS and Nisin, Farnesol and Polymyxin B Against Gram-Positive and Gram-Negative Bacteria. Curr. Microbiol..

[23] Cieslewicz M.J., Chaffin D., Glusman G., Kasper D., Madan A., Rodrigues S., Fahey J., Wessels M.R., Rubens C.E. (2005). Structural and Genetic Diversity of Group B Streptococcus Capsular Polysaccharides. Infect. Immun..

[24] Minnaganti V.R., Patel P.J., Iancu D., Schoch P.E., Cunha B.A. (2000). Necrotizing fasciitis caused by Aeromonas hydrophila. Heart Lung.

[25] FAO (2021). Risk Profile—Group B Streptococcus (GBS)—Streptococcus Agalactiae Sequence Type (ST) 283 in Freshwater Fish.

[26] Al-Bayati A., Douedi S., Alsaoudi G., Mosseri M., Albustani S., Upadhyaya V., Gornish N., Elsawaf M. (2020). Meningitis from invasive *Streptococcus agalactiae* in a healthy young adult. IDCases.

[27] Borrero J., Brede D.A., Skaugen M., Diep D.B., Herranz C., Nes I.F., Cintas L.M., Hernández P.E. (2011). Characterization of Garvicin ML, a Novel Circular Bacteriocin Produced by Lactococcus garvieae DCC43, Isolated from Mallard Ducks (Anas platyrhynchos). Appl. Environ. Microbiol..

[28] Maldonado-Barragan A., Cardenas N., Martïnez B., Ruiz-Barba J.L., Fernandez-Garayzabal J.F., Rodriguez J.M., Gibello A., Garvicin A. (2013). A novel class IId bacteriocin from *Lactococcus garvieae* that inhibits septum formation in *L. garvieae* strains. Appl. Environ. Microbiol..

[29] Tosukhowong A., Zendo T., Visessanguan W., Roytrakul S., Pumpuang L., Jaresitthikunchai J., Sonomoto K. (2012). Garvieacin Q, a Novel Class II Bacteriocin from *Lactococcus garvieae* BCC 43578. Appl. Environ. Microbiol..

[30] Villani F., Aponte M., Blaiotta G., Mauriello G., Pepe O., Moschetti G. (2001). Detection and characterization of a bacteriocin, garviecin L1-5, produced by *Lactococcus garvieae* isolated from raw cow’s milk. J. Appl. Microbiol..

[31] Dicks L.M., Dreyer L., Smith C., Van Staden A.D. (2018). A review: The fate of bacteriocins in the human gastro-intestinal tract: Do they cross the gut–blood barrier?. Front. Microbiol..

[32] Maher S., McClean S. (2006). Investigation of the cytotoxicity of eukaryotic and prokaryotic antimicrobial peptides in intestinal epithelial cells in vitro. Biochem. Pharmacol..

[33] Jasniewski J., Cailliez-Grimal C., Chevalot I., Millière J.-B., Revol-Junelles A.-M. (2009). Interactions between two carnobacteriocins Cbn BM1 and Cbn B2 from *Carnobacterium maltaromaticum* CP5 on target bacteria and Caco-2 cells. Food Chem. Toxicol..

[34] Varsha K.K., Nampoothiri K.M., Shilpa G., Priya S. (2021). Antimicrobial activity and cytotoxicity trait of a bioactive peptide purified from *Lactococcus garvieae* subsp. bovis BSN307 T. Lett. Appl. Microbiol..

[35] Hetz C., Bono M.R., Barros L.F., Lagos R. (2002). Microcin E492, a channel-forming bacteriocin from Klebsiella pneumoniae, induces apoptosis in some human cell lines. Proc. Natl. Acad. Sci. USA.

[36] Cox C.R., Coburn P.S., Gilmore M.S. (2005). Enterococcal Cytolysin: A Novel Two Component Peptide System that Serves as a Bacterial Defense Against Eukaryotic and Prokaryotic Cells. Curr. Protein Pept. Sci..

[37] Ravindran C., Varatharajan G., Rajasabapathy R., Sreepada R. (2016). Antibacterial Activity of Marine Bacillus Substances against *Vibrio cholerae* and *Staphylococcus aureus* and In Vivo Evaluation Using Embryonic Zebrafish Test System. Indian J. Pharm. Sci..

[38] Kalyanasundaram R., Radhakrishnan M., Anbarasu S. (2021). Activity and Toxicity Assessment of Partially Purified Bacteriocin from Lactic Acid Bacteria against Mycobacterium Kansasii. Ann. Rom. Soc. Cell Biol..

[39] Tseng C.-C., Murni L., Han T.-W., Arfiati D., Shih H.-T., Hu S.-Y. (2019). Molecular Characterization and Heterologous Production of the Bacteriocin Peocin, a DNA Starvation/Stationary Phase Protection Protein, from *Paenibacillus ehimensis* NPUST1. Molecules.

[40] Abriouel H., Franz C.M., Omar N.B., Gálvez A. (2011). Diversity and applications of Bacillus bacteriocins. FEMS Microbiol. Rev..

[41] Davis E.E., Frangakis S., Katsanis N. (2014). Interpreting human genetic variation with in vivo zebrafish assays. Biochim. Biophys. Acta (BBA)-Mol. Basis Dis..

[42] Phillips J.B., Westerfield M. (2014). Zebrafish models in translational research: Tipping the scales toward advancements in human health. Dis. Model. Mech..

[43] Patton E.E., Tobin D.M. (2019). Spotlight on zebrafish: The next wave of translational research. Dis. Model. Mech..

[44] Postlethwait J.H., Woods I.G., Ngo-Hazelett P., Yan Y.-L., Kelly P.D., Chu F., Huang H., Hill-Force A., Talbot W.S. (2000). Zebrafish Comparative Genomics and the Origins of Vertebrate Chromosomes. Genome Res..

